# Aggressive intentions after social exclusion and their association with loneliness

**DOI:** 10.1007/s00406-022-01503-8

**Published:** 2022-10-29

**Authors:** V. Brinker, J. Dewald-Kaufmann, F. Padberg, M. A. Reinhard

**Affiliations:** 1 Hochschule Fresenius, University of Applied Sciences, Munich, Germany; 2 Department of Psychiatry and Psychotherapy, LMU University Hospital Munich, Munich, Germany

**Keywords:** Perceived social isolation, Ostracism, Social rejection, Social connection

## Abstract

Both social exclusion and loneliness are aversive experiences that can lead to hostile behavioral reactions, including aggressive behavior. This study aimed to assess whether a social exclusion scenario in the subject’s imagination elicits aggressive reactions towards an excluding person as measured with the hot sauce paradigm. Furthermore, we studied the effect of loneliness on such reactions. In total, 251 subjects (67.7% female; mean age 27.3 ± 9.3 years) participated in this study which was based on an online survey. After trait loneliness was assessed with the UCLA Loneliness scale at baseline, two imaginary scenarios were presented in randomized order, i.e., an exclusion condition (with one of two working colleagues excluding the participant from a social activity) and an inclusion condition (without exclusion). Following each scenario, participants had the task to allocate the amount of hot sauce to each colleague that they find appropriate. Participants distributed significantly more hot sauce to the excluder than to the includers. The amount of hot sauce was significantly correlated with loneliness for all includer interactions (i.e., after the inclusion as well as the exclusion scenario), but not for the interaction with the excluder. Our results support the hypothesis that social exclusion elicits aggressive behavior. Interestingly, the experience of loneliness seems to be associated with an increase in aggressive behavioral tendencies or a lack of their inhibition. The cognitive and/or emotional processes underlying the interplay between social exclusion, loneliness and aggression should be a focus of future research.

## Introduction

Social belonging and connectedness are inert human needs [1] that can negatively impact people’s mental well-being. When this need to belong is not sufficiently met, the feeling of loneliness can arise [2]. Defined as the discrepancy between actual and desired levels of social interaction [3], loneliness may motivate reconnection with others. Yet, loneliness often persists due to different reasons such as social withdrawal [4], hypervigilance to social cues of rejection and negative cognitive biases [5]. Furthermore, loneliness has been associated with hostility or even aggression [6, 7], that may elicit rejection by others and thus increase social isolation and loneliness.

Situations of social exclusion and rejection are a particular threat to the above-mentioned need to belong and of particular interest in the context of loneliness. The term social exclusion refers to a person’s experience of being excluded from a group of other individuals [8]. Common experimental approaches to assess the effects of social exclusion are (virtual) real-time interactions like the Cyberball paradigm (a virtual ball-tossing game that excludes the individual), negative feedback paradigms or imagined scenarios [9, 10]. Typically, social exclusion negatively affects emotional and behavioral reactions. According to the need-threat model the perception of social exclusion leads to an immediate reaction such as social pain, negative emotions (e.g., sadness, anger) and threatened intrapersonal needs such as the need to belong and to maintain control [11, 12]. Following this reflexive stage, the excluded individual responds with behavioral actions. As Smart Richman and Leary [13] propose, reactions vary from prosocial behavior to withdrawal or even antisocial behavior and aggression [14].

The specific behavioral reaction to the experience of social exclusion may depend on various factors. In the case of a predisposition for aggression, factors like narcissism, rejection sensitivity and hostile beliefs about interpersonal relationships have been suggested [15]. Accordingly, Ayduk et al. [16] used the classical “hot sauce” paradigm of aggressive behavior [17, 18] and observed that individuals with high rejection sensitivity (RS) showed greater retaliatory rejection (i.e., giving less favorable impression ratings to the other person) and allocated more hot sauce to a person who purportedly rejected them as a chat partner than low RS individuals. Similarly, the presence of loneliness may lead to a more aggressive reaction when being socially excluded. However, most of the prior studies on loneliness and aggression measured aggression with questionnaires that assess aggression in general [19–21] and not in the ecologically more valid context of specific situations such as social exclusion.

Thus, this study investigates whether the imagination of a social exclusion scenario may elicit aggressive reactions towards the excluder as measured with the hot sauce paradigm. Further, we hypothesized that loneliness is associated with increased aggressive reactions towards the putative excluder compared to including persons.

## Method

### Participants

Participants were recruited through the University’s internal service portal as well as social media platforms (e.g., Facebook, Instagram, Survey Circle platform) and took part in an online survey between March 20th and May 5th 2021, which was not limited in sample size beforehand. Inclusion criteria were a minimum age of 18 years and sufficient German language skills. The study was approved by the local Ethical Committee of the Hochschule Fresenius, University of Applied Sciences, Munich, Germany, and was performed in accordance with the ethical standards laid down in the Declaration of Helsinki. After checking the inclusion criteria, participants had to give written consent.

### Procedure

From all participants, sociodemographic data were obtained, and loneliness was assessed with the UCLA Loneliness scale [22]. Afterwards they were asked to imagine two interpersonal scenarios with colleagues at work, which were presented in randomized order (i.e., randomized controlled cross-over study; simple random allocation of order performed by the online platform). In scenario 1 (the inclusion condition), participants were told they work with two colleagues, who integrate the participants into their interactions. In scenario 2 (the exclusion condition), participants were told by one of the two colleagues that their presence was not appreciated in a joint social activity. After each scenario, participants were asked to allocate hot sauce to both colleagues separately. Further, participants had to express on a five-point Likert Scale how much they could empathize with the specific scenario (1 = very rarely, 5 = very well). After the experiment, participants were thanked and debriefed. Participants had the chance to win one out of five Amazon vouchers (€25 each). Psychology students were offered 0.5 study credit hours for participating.

### Instruments

#### Aggression

The Hot Sauce Paradigm is a well-established behavioral paradigm to measure aggressive behavior and was used as a primary outcome in this study [17, 18]. Participants were asked to allocate a certain amount of hot sauce to a target person. The amount of hot sauce represents a behavioral index of the level of aggression (0 g = no sauce up to 2000 g = maximum amount of sauce).

#### Loneliness

The German version of the revised UCLA Loneliness Scale was used to measure trait loneliness [22, 23]. Participants were asked to rate 20 items (e.g., I am feeling comfortable when I have people around me) on a five-point Likert Scale (1 = does not apply at all, 5 = does apply). An overall score was calculated with higher scores indicating greater loneliness. The internal consistency in this sample was high (Cronbach’s* α* = 0.94).

### Data analysis

Statistical analyses were conducted with SPSS version 25. Data is presented in means and standard deviation. As the allocation of hot sauce was not normally distributed, a non-parametric Friedman test was performed with Dunn-Bonferroni tests as post-hoc tests to compare the allocation of hot sauce to the different colleagues. Finally, Spearman correlations were used to calculate the association between loneliness and the allocation of hot sauce.

## Results

### Sample

The sample consisted of *N* = 251 participants (170 female, 67.7%) with a mean age of 27.3 ± 9.3 years and mean years of education of 16.8 ± 3.3 (secondary school, “Abitur”: *n* = 105, 41.8%; university degree: *n* = 122, 48.6%; primary school: *n* = 6, 2.4%; vocational training: *n* = 18, 7.2%). The majority of participants was in a relationship (*n* = 124, 49.4%) or married (*n* = 28, 11.2%) and *n* = 97 (38.6%) were single or divorced (*n* = 2, 0.8%). Most participants were living together with family or family members (*n* = 101, 38.2%), with their partner (*n* = 72, 28.7%) or with flatmates (*n* = 32, 12.7%). *N* = 46 (18.3%) were living alone.

Mean loneliness score was 2.0 ± 0.7. Men and women did not differ regarding levels of loneliness (*t*[138.7] = 0.42, *p* = 0.68). Further, age showed no significant correlation with loneliness (*r*_*s*_ = -0.08, *p* = 0.20).

### Hot sauce after exclusion/inclusion

Participants reported high levels of empathy with both scenarios (exclusion: mean 4.0 ± 0.9; inclusion: mean 4.2 ± 0.7). The average use of hot sauce ranged from 100.0 ± 300.4 to 105.3 ± 314.9 in the inclusion condition and from 155.6 ± 330.6 (including colleague) to 349.4 ± 555.0 (excluding colleague) in the exclusion condition.

**Fig. 1:**
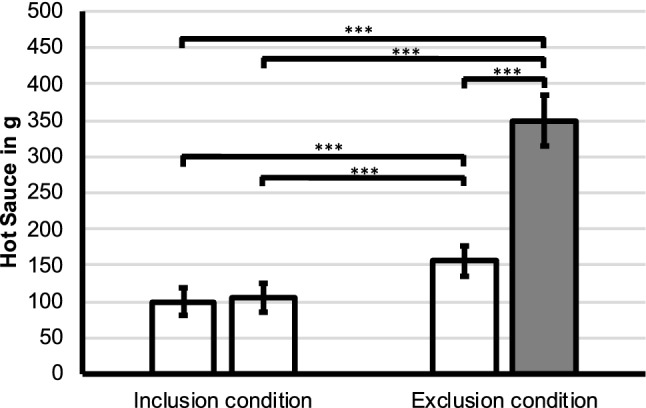
Hot Sauce allocation to the including (white bars) and excluding person (gray bar), please note that only one colleague actually excluded the participant in the exclusion condition of the paradigm. Significant differences are indicated as ***p < 0.001 (Bonferroni-corrected)

We found a significant difference in the amount of hot sauce distributed to the four colleagues in both conditions (*χ*^2^[3] = 218.29, *p* < 0.001, *n* = 251). Post-hoc tests revealed that participants particularly distributed hot sauce to the excluder (see Fig. 1) as compared to the includer in the exclusion condition and compared to both including persons in the inclusion condition (all pairwise comparisons: *z* > 0.63, *p* < 0.001). Further, the includer in the exclusion condition received more hot sauce than both includers of the inclusion condition (both: *z* > 0.45, *p* < 0.001). No difference was found between both persons in the inclusion condition (*z* = 0.06, *p* = 0.99).

Men and women did not differ regarding the distribution of hot sauce to the four colleagues (all *t*[249] < 1.48, all *p* > 0.14) and age showed no significant association with the use of hot sauce (Spearman correlation, all *r*_s_ < 0.07, all *p* > 0.30).

### Loneliness and hot sauce

**Fig. 2:**
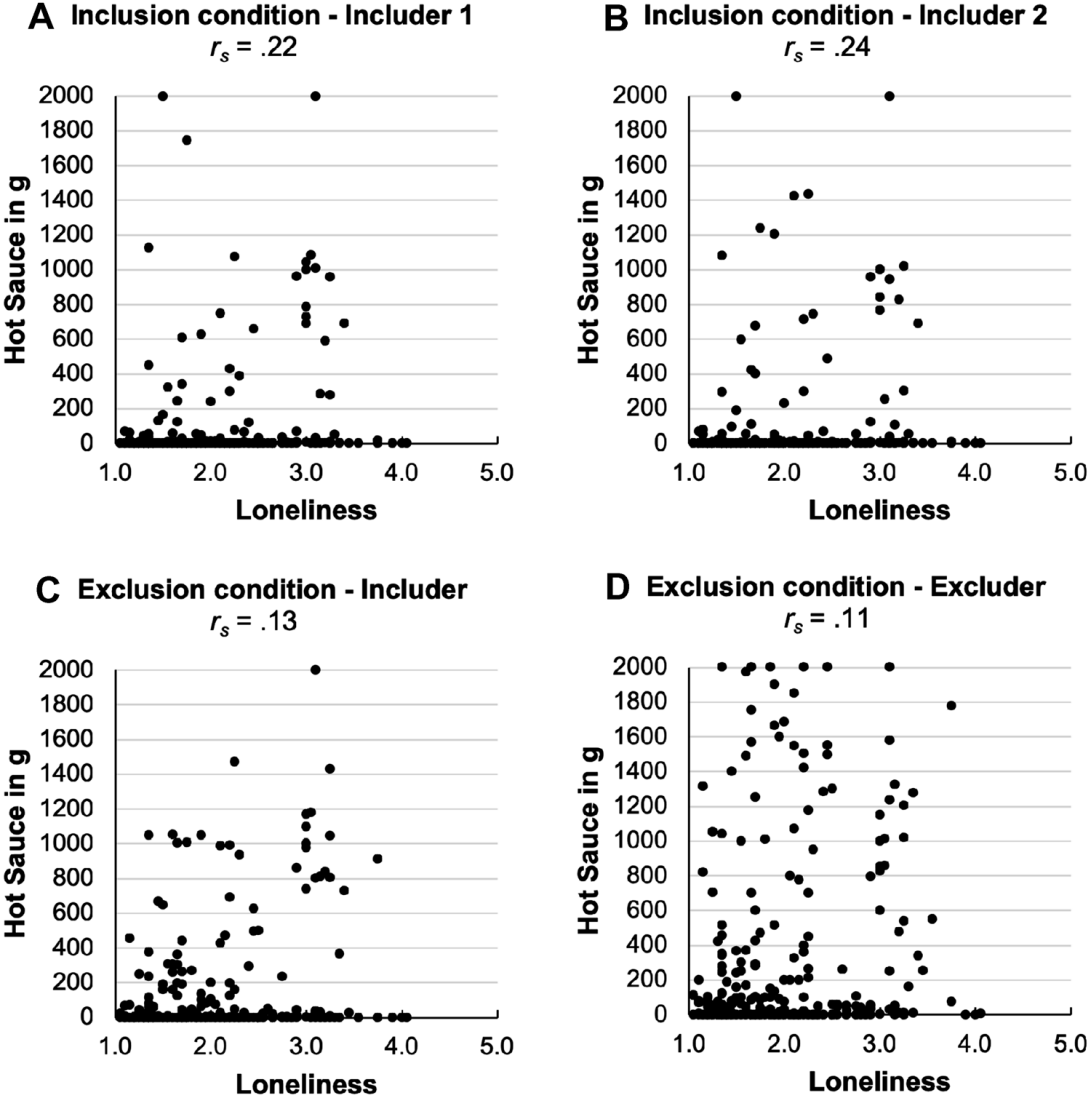
Scatter plots of Loneliness and Hot Sauce allocation for the three including colleagues (**A**, **B**, **C**) and the excluding colleague (**D**; *r*_s_ = Spearman correlation)

The UCLA loneliness scores were significantly associated with more hot sauce allocation to the respective includer both in the inclusion (Spearman correlation, *r*_s_ = 0.22 and *r*_s_ = 0.24, both *p* < 0.001) and the exclusion condition (*r*_s_ = 0.13, *p* = 0.04), but not to the excluder (*r*_s_ = 0.11, *p* = 0.07; see Fig. 2).

## Discussion

The main finding of this study was that the imagination of a social exclusion scenario led to aggressive intentions in the hot sauce paradigm, i.e., the distribution of hot sauce to the excluder compared to inclusion conditions. Interestingly, the amount of hot sauce in all inclusion conditions, i.e., towards the including person in the exclusion condition as well as towards both persons in the inclusion condition was associated with the UCLA loneliness score at baseline, whereas for the excluder this association was not observed.

An association between loneliness and aggression has been described before as early as 1938 [21, 24] and has been reported in several studies that mainly assess aggression with self-report questionnaires on a more general level in adults [19–21]. In addition, lonely children were found to be more aggressive towards others [25, 26] and childhood loneliness predicted later aggression in youth [7]. Finally, one study [6] used a behavioral measurement for aggression and found that lonely males not only expressed more hostility but reacted more aggressively towards a rejecting confederate.

Contrary to our hypothesis, loneliness was associated with increased allocation of hot sauce especially to the including colleagues compared to the excluding colleague. Whereas the experience of rejection may generally lead to more aggressive behavioral tendencies, the presence of loneliness may predispose one to react more aggressively to others who are actually not excluding this person when actually being included. This may be explained by previous findings that lonely individuals nevertheless expect to be rejected [5, 27], have a general negative opinion of others, perceive others’ intentions more negatively and are more sensitive to cues of social exclusion [28]. Alternatively, the effect may be explained by low empathy that seems to be associated with loneliness [29]. Lonely individuals may have deficits in recognizing and interpreting others’ emotions and behavioral reactions [29], which may disinhibit aggressive behavioral reactions.

As aggressive reactions to social exclusion may lead to further exclusion, rejection and loneliness, possible exits of this vicious circle need to be identified. Therapeutic interventions may focus on specific target points in this circle. For instance, the expectancy of being rejected could be addressed with cognitive reappraisal strategies, role plays and behavioral experiments. Further, lonely individuals may need to increase their social self-efficacy by learning strategies on how to restore control and increase predictability in social relationships. However, loneliness is often not assessed or mentioned in the clinical setting but may represent an important trait or symptom associated with chronicity and suicidality in mental health conditions [30, 31]. Clinicians should be aware that loneliness may represent an underlying factor when being faced with aggressive intentions in the absence of other obvious reasons for aggression (such as social exclusion and rejection). Consequently, our findings may be particularly relevant regarding psychiatric disorders where loneliness is common such as personality disorders ([32], e.g., borderline personality disorder) or depression.

Future research may need to investigate the interplay of loneliness and aggression in specific clinical samples (e.g., depression). In addition, loneliness may be incorporated into anti-aggression interventions to increase their effect. Finally, basic research on cognitive and/or emotional processes underlying the interplay between social exclusion, loneliness and aggression is warranted including experimental designs that manipulate the intensity of loneliness.

Strength of our study is the ecological validity of our imagined scenario in a population-based sample. However, several limitations need to be mentioned: First, UCLA loneliness scores were only measured as a trait at baseline but may have been affected by the imagined scenarios. It would be of interest to measure state loneliness before, during and after the imagined scenario and after hot sauce allocation. Second, there is a lack of a more elaborate experimental design, e.g., measuring felt control and rejection expectancy for the different imagined scenarios. Third, a more detailed assessment of inter-individual differences between participants may have further explained which factors contribute to aggressive reactions (including the assessment of any psychiatric history or diagnosis).

To conclude, imagined social exclusion was sufficient to elicit an aggressive reaction especially towards the excluding colleague. Surprisingly, the magnitude of aggressive tendencies was associated with loneliness at baseline across all includer interactions (i.e., after the inclusion as well as the exclusion condition). Understanding the mechanisms of how loneliness, social exclusion and aggression maintain each other, may enable us to develop therapeutic interventions for this vicious circle of dysfunctional interaction.
